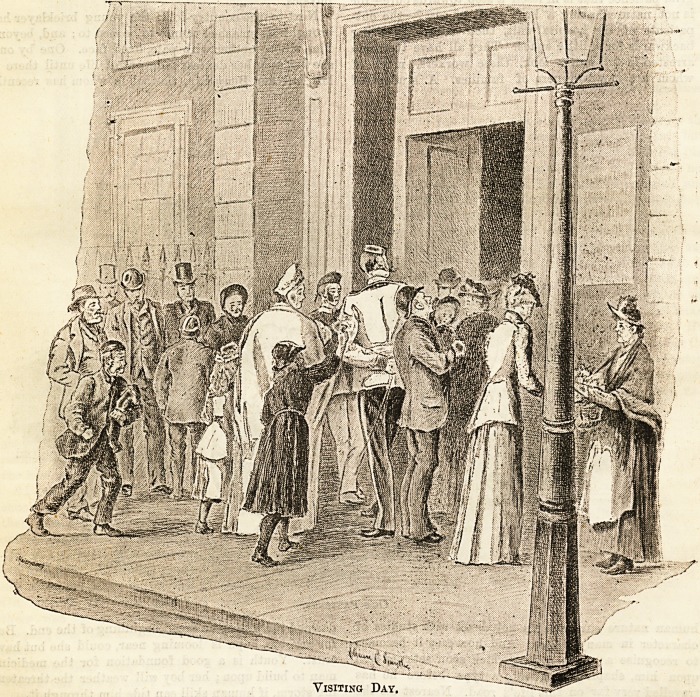# A Live-Long Day

**Published:** 1891-05-30

**Authors:** 


### For Hospital Sunday Readers, 1891.

### A LIVE-LONG DAY.

Be the day weary or be the day long,
At length it ringeth to even-song.

### Dawn.

It was the grey dawn, just when night and day were greeting each other. In the ward the occupants of the white beds were wakeful, for a subtle sense had

The Ward Sister.

penetrated their dreams of a new, strange presence among them.

"It's terrible quiet over yonder!" went the whisper from one bed to another.

"Over yonder" was the corner where the screen was drawn round a bed, hiding a human tragedy. A mother, young and fair, but spent with disease, was sobbing out her farewell to the children she must leave on earth. On her knees, close by, knelt Sister Irene, murmuring of Him to whom the dying cling.

"I know, I know, Sister; I try to trust. But it's hard to say good-bye to my little ones," was the strangled answer.

"Have no fear; we shall care for *them*. And it's but for a little while; you'll be standing close to the gates of gold, peering through to watch them toiling up the steeps of earth, and think of the joy of that meeting!" And the peace of that blessed hope smoothed the anguished face, and brooded round the sweet lips, but not yet was the little mother to slip into the arms of the angel waiting to bear her afar off; God is very good, very patient. Not yet, were the trembling, stricken ones to be left motherless and lonely. Every day science opens new doors, and the last tried treatment is suddenly successful. With a soft rustle of its wings the angel of death withdraws. Slumber, God-sent and healing, instead of death, steals over the patient behind the screen. Then the Sister, whose name is Peace, rises, and leads the little ones away through the dim ward out into the light of a new life all aglow with hope, whispering to them as they go the blessed news that, "All is well; mother will get better now!"

### Morning.

Nine o'clock has struck. The night nurses have melted away to rest, and, in their stead, reign the day nurses. Luncheon has been served, some of the patients having to be fed with tender patience; then comes a lull of waiting before the doctor's advent. Stretched out still and motionless lies a fair-faced woman, the short curls pushed up from her broad white brow; she is partially paralysed from a terrifying shock. Bending over her is the masseuse whose lissom fingers are busy trying to coax restoration back to the vital functions.

"Shall I ever walk again, Allie?" asks a harsh, constrained voice, and Nurse Alice lifts a startled face. In her rapt anxiety over her work she had scarce glanced at the features of the new patient; and now she sees before her one who long since stepped in between a dear dead sister and her life's happiness, blotting out its sunshine.

"So you didn't know me, Allie, then?" goes on the jangled voice. "I remembered *you* at once; we never forget those we injure, and poor dead Katie's memory haunts me ever. But it has all come home to me, Allie; he deserted me—left me for a newer face, and I lost heart. I have slipped down, down, until I earn my bread slaving with the embroidery needle; and the fire at the lodging-house brought me here. Are you *glad?*"

Ward by Day—Massage.

For answer, Nurse Alice's lips were pressed silently to the wan face of her enemy; it was the seal of a good woman's pardon—a touch of moral massage to heal and soothe the rankling of sin.

A Small Patient's Dinner.

Noon.

"I can't!" wailed a hopeless little voice, a voice lacking all childish glee; "I want Daddy. I'm Daddy's pet, and I'll let him feed me."

"But won't you play at being *my* pet just for this once, and let me give you your dinner?" and the owner of a pure, earnest face seated herself on the cot, the bowl of soup in her hand.

"I don't know *you*!" The tiny head shook mournfully from side to side; then, the wee sufferer's attention was suddenly arrested. "Did you buy your eyes at a shop? Mother's eyes are so old and dim. Yours are shiny, like the new sixpence in my money-box at 'ome. Oh, where's my 'ome gone to?" and there was an uncontrollable burst of weeping over the all-at-once discovered loss.

"Hush, dear one, hush-h! Listen, while I tell you a story about twenty little fairy maids who once sat round the brim of this bowl swinging their feet to and fro; there was one who was naughty, and wouldn't eat her dinner—and so she fell in! Suppose we eat up the soup, just to see if we can find her at the bottom!"

Little Misery walks straight into the pretty trap; the spoonfuls disappear; then, lo! smiles chase away the tears when at the bottom of the bowl is found the picture of little Miss Muffet sitting beside her Spider.

Midnight.

Outside, night looks down with her "thousand eyes," upon the city's secrets. The hour of midnight is long past, but the going to and fro of many feet upon the pavement is little less than at noon-day. Indoors, there is, throughout the ward, a restful atmosphere of peace and purity. By the light of the shaded lamp Nurse Eleanor writes down notes in her case-book. From a distant bed a moan breaks forth, and she noiselessly moves to the side of the wakeful sufferer. It is the stalwart young trooper dashed down, yesterday, by the hansom, from the wheels of which he sprang to snatch a child who had, with uncanny swiftness, darted from its mother's side to stagger, all innocent of danger, into the congested mass of traffic.

"I *can't* sleep!" whispers the feverish soldier, smothering another groan.

"Should you like to talk about home?" suggests the soft voice, thinking to relieve the excited brain; but, instead, he tells her of India, from which he has just come, tells queer stories over which she knows not whether to laugh or to cry, but his mind is rested and pacified, and, presently, a lull steals over him, but sleep comes with tardy steps.

"I'm main glad you've come, Jenny, dear," he dreamily says, taking the nurse, between waking and sleeping, for his sweetheart; "it's all settled; we're to be married at once. I've spoken to my captain and got his promise, so you'll be on the strength of the regiment, dear," and lifting Nurse Eleanor's cool hand, he suddenly places it across his fevered brow. His thoughts are far away; he is by Jenny's side under the summer-show of the orchard trees in the home country, and it is Jenny's hand. A flush dyes the woman's pure, fair face, but the *nurse* remembers in time that any

The Ward at Night.

sudden jar may send Trooper H. down the wrong road, and she lets her hand rest there—for Jenny's sake.

### Fallen Out by the Way.

Through the streets of the mighty city goes on the ceaseless tramp of life's monotonous march. But now and again one or another drops out of the ranks, struck down by accident or by stealthy disease, and stumbles away to that haven he wots of to have his wounds healed—to be set up again, as he phrases it. With a grim patience that is in itself a step towards cure—for is not nature having a brief spell of rest?—the out-patients of the hospital wait, their minds travelling backwards for a little space. They all have the same arrested-in-full-swing look, these workers, most of whom are bread-winners of families. A student of human nature enriches his note-book with studies of character in many phases. And how easy it becomes to recognise a man's calling which soon sets its seal upon him, shaping him into a type before he has travelled very far on this life's road. Nearest on the bench sits the unmistakeable coster, who, finding his rheumatics more than he can well bear, comes, full of faith, for a trifle of doctor's stuff to put him right, albeit grudging the precious moments wasted on those old, aching bones of his, and brooding moodily over what "that boy is a-doing with the donkey and the cargo of vegetables" in his absence.

The couple next have a graver cause for anxiety; he has braced himself to seek and to hear the verdict about "the old complaint," which is getting too masterful of late; and she, the faithful wife, has come to back him up if the news should be bad. The coming up to London for the best advice has been a long-talked-of event achieved at last; the journey has been weary and bewildering, but above the pair hangs the sweet breath of the country as well as its slow ways. Courage, old man! There is skill of the highest awaiting you within, ready to ease your heart of its haunting burden, and set you free—free to return to the shady lanes and flowery meads, with a light heart, and a solemn reverence for "they London doctors and their physics."

Next to the country-folk, the young bricklayer has brought his smashed thumb to be seen to; and, beyond him, sits a mother with pale, fixed face. One by one, she has seen her dearest fade out of life until there is left but her Benjamin, the lad in whom has recently dawned the hint—the dread beginning of the end. But for her, too, hope is looming near, could she but have trust. Youth is a good foundation for the medicine man to build upon; her boy will weather the threatening storm, if human skill can tide him through it.

Out Patients.

A brighter side to the story of suffering, written so plainly for seeing eyes on each and every face in the hall, is the old cobbler, with his crutch, in the back row; the cheery old man, who wisely looks on the sunny aspect of all things, even of pain. He is not familiar with Browning's grand line—

God's in heaven: all's well on the earth, but that deep-seated conviction is the secret spring of his placid content as he waits his turn—waits as did the impotent folk of old for "the troubling of the water."

### Visiting Day.

The red-letter day at last, and the doors are opening. "Buy a bunch, miss? Which will you 'ave?" says the flower-girl, sure of her harvest among the eager crowd, for who would pass a posy on his way to visit the sick?

"Give me lilac," says the pretty girl who is going to see her sweetheart, the poor young city clerk whom the influenza and its train of ills have bowled over; "give me lots of it!" and she whispers to herself that *he* will remember it was under the lilacs by the gate of the old home that their troth was plighted.

Visiting Day.

"Be good now, baby, and we'll soon see mammy!" enjoins the father, holding tighter the tiny maid, who gives a crow of expectant delight. Near them a young soldier towers over all heads; he is going to see his chum—a street accident case—and another flower-girl presses behind him, holding aloft her proffered roses. And here comes, all hurry and eagerness, a street arab, with some hardly-got treasure for the cripple sister. One and all, the crowd is bent on the same mission.

Upstairs, there is a simmer of expectation in the wards; everybody is prepared in readiness, and for each visitor coming up a heart is beating a welcome. How eager the thirst to know what Johnny is doing, and if father's keeping steady, and if baby cries much for mother! The hospital-world is made up of such "touches of nature," and is ever ready to be "kin."

By-and-by, when the sun of the visiting day is set and it is even-tide, the nurses will have to play listeners, and hear from bed and cot each occupant's news from the outside world. One will have to be shown, and be quick to admire Cripple Molly's new doll, which brother Jem's self-denied halfpence have managed to buy; unlovely and cheaply robed, but a peerless doll in the eyes of both Molly and the proud street arab.

And some one else must see and praise that bunch of roses the soldier's chum brought in, half ashamed of such a womanish action.

"Couldn't you give 'em to yon poor little lass over there?" pants the patient, and Molly, richer than ever, kisses, with a pathetic little cry, the welcome roses.

Happy chatter, counting over treasures, dreams of a good time coming, all pass; and then over the wards steals the hush of restful night.

## Figures and Tables

**Figure f1:**
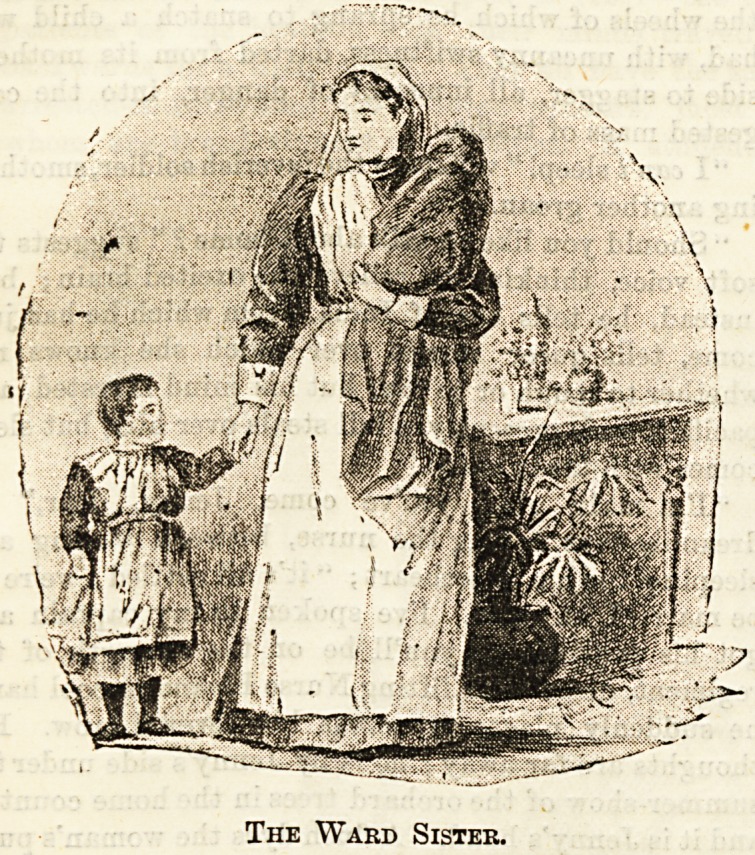


**Figure f2:**
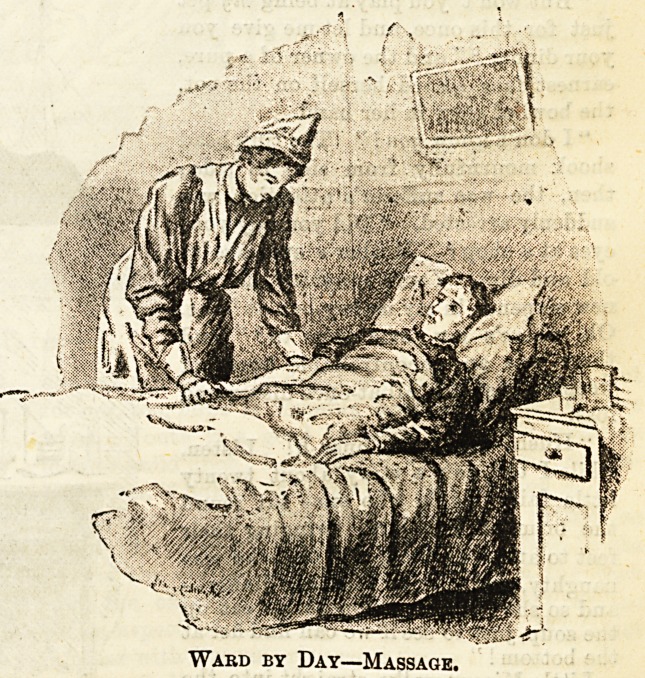


**Figure f3:**
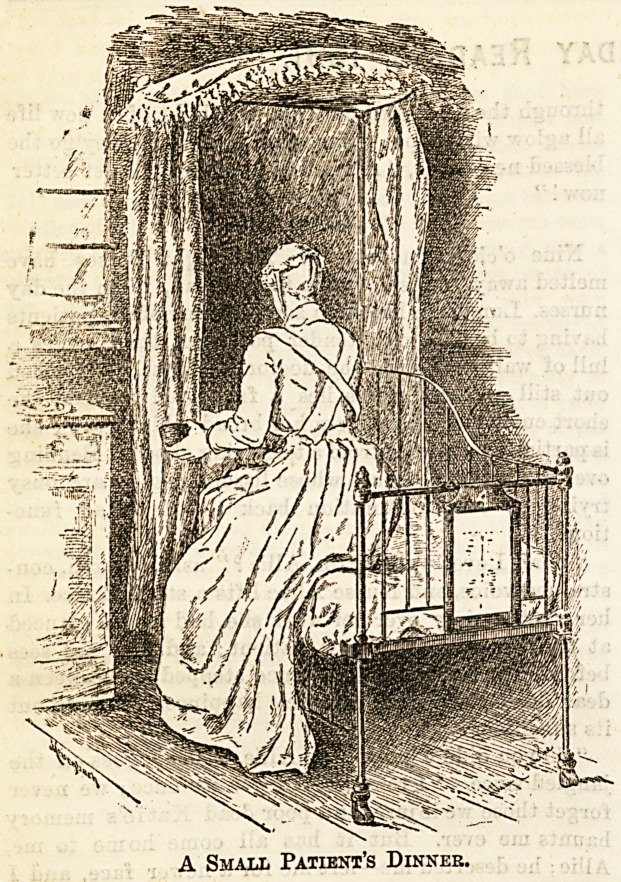


**Figure f4:**
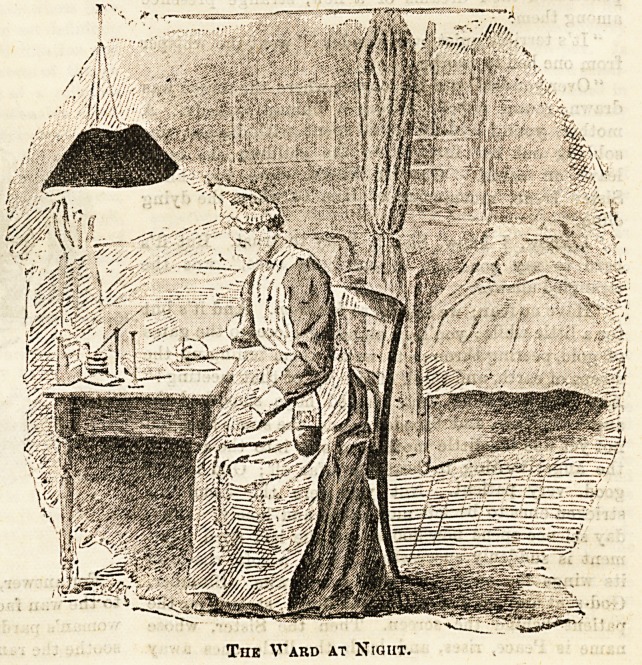


**Figure f5:**
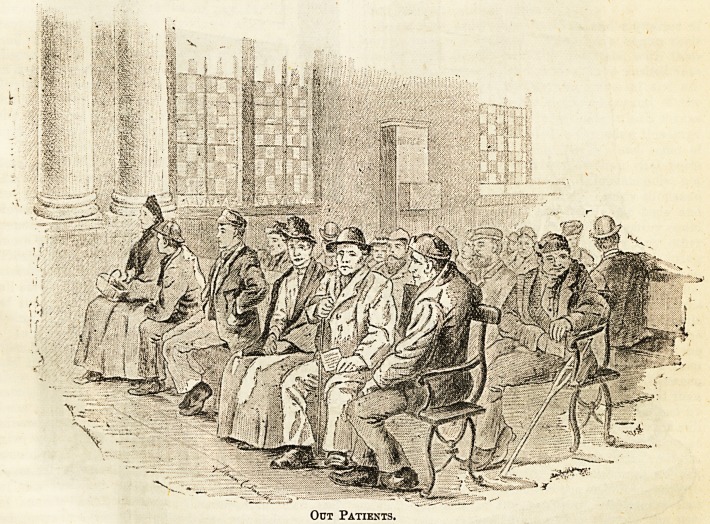


**Figure f6:**